# Investigation of the Combined Influence of Temperature and Humidity on Fatigue Crack Growth Rate in Al6082 Alloy in a Coastal Environment

**DOI:** 10.3390/ma16216833

**Published:** 2023-10-24

**Authors:** Ibrahim Alqahtani, Andrew Starr, Muhammad Khan

**Affiliations:** Centre for Life-Cycle Engineering and Management, School of Aerospace, Transport and Manufacturing, Cranfield University, College Road, Cranfield MK43 0AL, UK

**Keywords:** Al-Mg-Mn-Si alloy, fracture toughness, coastal environments, polynomial model, failure mechanism

## Abstract

The fatigue crack growth rate (FCGR) of aluminium alloys under the combined influence of temperature and humidity remains a relatively unexplored area, receiving limited attention due to its intricate nature and challenges in predicting the combined impact of these factors. The challenge was to investigate and address the specific mechanisms and interactions between temperature and humidity, as in coastal environment conditions, on the FCGR of aluminium alloy. The present study conducts a comprehensive investigation into the combined influence of temperature and humidity on the FCGR of the Al6082 alloy. The fatigue pre-cracked compact tension specimens were corroded for 7 days and then subjected to various temperature and humidity conditions in a thermal chamber for 3 days to simulate coastal environments. The obtained data were analysed to determine the influence of temperature and humidity on the FCGR of the Al6082 alloy. An empirical model was also established to precisely predict fatigue life cycle values under these environmental conditions. The correlation between FCGR and fracture toughness models was also examined. The Al6082 alloy exhibits a 34% increase in the Paris constant C, indicating reduced FCGR resistance due to elevated temperature and humidity levels. At the same time, fatigue, corrosion, moisture-assisted crack propagation, and hydrogen embrittlement lead to a 27% decrease in threshold fracture toughness. The developed model exhibited accurate predictions for fatigue life cycles, and the correlation between fracture toughness and FCGR showed an error of less than 10%, indicating a strong relationship between these parameters.

## 1. Introduction

Aluminium alloys in the 6xxx series have gained significant popularity and are widely utilised in diverse structural applications such as marine applications [1], rotor blades, and aircraft wings [2]. Aluminium has found extensive application in marine environments [3,4] due to its unique properties, including its light weight, corrosion resistance, and mechanical strength. However, in coastal conditions, where there are temperature and humidity variations [5], factors such as corrosion [6], fatigue loading [7], fatigue crack growth rate (FCGR) and fracture toughness [8,9,10,11] can affect its performance.

The connection between FCGR and fracture toughness is essential in understanding the fatigue behaviour of aluminium alloys [12]. Various deterministic FCGR functions, including the Paris–Erdogan [13], Walker, Trantina–Johnson, Forman, and generalised Forman models, have been proposed and widely utilised in the literature [14]. A material with high fracture toughness typically exhibits a slower FCGR, indicating better resistance to crack growth under cyclic loading. Understanding crack growth behaviour and the factors influencing it is paramount for confirming the structural integrity and reliability of components subjected to repeated loading. Materials are often exposed to complex environmental conditions [15] that significantly affect fatigue performance in real-world applications [5,16]. Among these conditions, temperature and humidity variations, particularly in coastal environments, play a critical role in finding the extent of crack growth and the overall durability of materials [5]. However, the fatigue behaviour of the aluminium alloy under combined exposure to temperature and humidity in coastal environments is poorly understood.

The coastal environment, characterised by high humidity levels and fluctuating temperatures, poses unique challenges to materials due to the corrosive nature of the atmosphere [14]. Middle Eastern locations can experience summertime temperatures close to 60 °C [17], high humidity levels ranging from 40% to 90%, and a chloride ion concentration of approximately 3.6% [17,18]. The heat generated by components during their operating condition can exceed 70 °C, impacting their performance. In these circumstances, structures can reach temperatures of up to 80 °C, with humidity levels of 90% [5]. The presence of chloride ions, moisture, and temperature variations can accelerate corrosion processes, leading to increased crack growth rates and reduced fatigue life.

Several factors, including corrosion, temperature, and humidity, influence the aluminium’s FCGR. Corrosion can lead to localized weakening of the material, accelerating crack growth. Temperature variations can affect the diffusion of atoms along the crack front, influencing crack growth rates. Additionally, higher humidity levels can promote crack propagation due to moisture and environmental factors.

The correlation between FCGR and fracture toughness is critical in understanding the fatigue behaviour of the Al6082 alloy. Fracture toughness represents the material’s capability to resist crack propagation and absorb energy before failure. Higher fracture toughness values indicate better crack resistance. Meanwhile, FCGR measures the rate at which cracks extend under cyclic loading. The FCGR is influenced by factors such as stress levels and crack size.

The Paris model, also known as the Paris Law or Paris–Erdogan Law, is a mathematical relationship used to predict the growth of fatigue cracks in materials over time. Three distinct regions are observed in a da/dN vs. ΔK plot. The threshold region, characterized by low ΔK values, shows minimal crack growth due to closure effects. The Paris region, with intermediate ΔK values, displays a linear connection between da/dN and ΔK, representing stable crack growth. At high ΔK values beyond the Paris region, the accelerated growth region significantly increases da/dN, leading to failure [19].

In coastal conditions, environmental factors such as temperature and humidity impact the corrosion FCGR of aluminium alloys. Hassaan Bin Younis et al. [20] utilized three specialised optimised neural networks to forecast the rate of fatigue crack growth (FCG) under the direction of a mutated leader algorithm (MLA). These methods make use of optimised neural networks based on genetic algorithms, hill climbing optimisation techniques, and simulated annealing optimisation algorithms. Testing on a variety of aluminium alloys that are often used in the aerospace sector is undertaken to validate the algorithms described in this novel technique. Danyil Kovalov et al. [21] explored corrosion fatigue in aluminium alloy 2024-T351, finding influence from electrochemical potential, NaCl concentration, loading frequency, and temperature. Tianyu Zhang et al. [22] developed a model accounting for corrosion and fatigue alternation effects on 2024-T4 aluminium alloy.

However, despite utilising various models [21,22,23] to analyse the obtained data and predict the life cycle under both non-corroded and corroded conditions, the specific effect of temperature and humidity remains unaddressed in the current understanding. Investigating the combined impact of temperature and humidity on FCG behaviour is still critical for ensuring the reliable and safe performance of components made from the Al6082 alloy in coastal environments. Previous studies have separately examined the influence of temperature [24,25,26,27] and humidity [28,29,30] on the fatigue properties of materials [31,32,33,34]. However, a comprehensive understanding of the combined impact of these factors is critical for accurately predicting fatigue life [35] and developing empirical equations for predicting and correlating FCGR with fracture toughness.

This research aims to address this knowledge gap by studying the Al6082 alloy’s FCGR under controlled temperature and humidity conditions that simulate a coastal environment. This study focuses solely on investigating the stable crack growth regime in the da/dN vs. ΔK plot. The research is limited to examining crack growth behaviour within this region, where crack growth is predictable and stable. The influence of temperature and humidity variations on the crack propagation characteristics will be evaluated by subjecting the alloy to cyclic loading and monitoring crack growth. The novelty of this work lies in the thorough investigation of FCGR under combined temperature and humidity conditions in a coastal environment, the development of an empirical model for accurate fatigue life prediction, and the correlation between FCGR and fracture toughness models. These contributions significantly advance the understanding of the fatigue behaviour of the Al6082 alloy in coastal environments, leading to a comprehensive understanding of aluminium alloy for potential applications in coastal environments.

## 2. Methods

### 2.1. Material

The Al6082 alloy is well-known for being strong and lightweight, making it popular in the aerospace, automotive, and marine industries [36]. Table 1 presents the main elements present in the Al6082 alloy. The Al6082 alloy has a density of 2.71 g/cm^3^, elastic modulus of 70 GPa, yield strength of 320 MPa, tensile strength of 348 MPa, and elongation of 17.5%. Its fracture toughness is 28.63 MPa√m in non-corroded conditions and 23 MPa√m in high humidity conditions at room temperature [5].

### 2.2. Specimen Preparation

**Figure 1:**
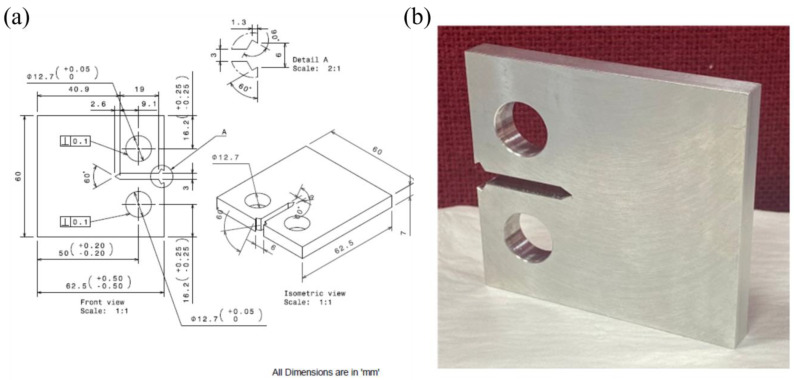
Specimen: (**a**) Geometry and (**b**) prepared sample.

The preparation of the compact tension (CT) specimen from a 7 mm thick sheet of Al6082 alloy was undertaken following the specified geometry, as depicted in Figure 1 [37]. Wire-cut EDM was used to cut the notch in the specimen’s centre [38]. The CT specimens underwent pre-cracking using a servo-hydraulic testing apparatus. Additionally, a fatigue crack of 3 mm was introduced at the notch’s end for all CT specimens, ensuring the maintenance of a consistent a/W (crack length-to-width) ratio [39]. By incorporating a fatigue crack before conducting the FCGR test, the focus shifts from an evaluation of the material’s strength or ductility to an emphasis on its ability to endure crack propagation and fracture. This distinction is significant as each material inherently possesses very tiny, sharp cracks [40]. However, it is essential to note that the notch created using wire-cut EDM may not accurately replicate the characteristics of cracks within the material.

### 2.3. Simulation of Coastal Environment

**Figure 2:**
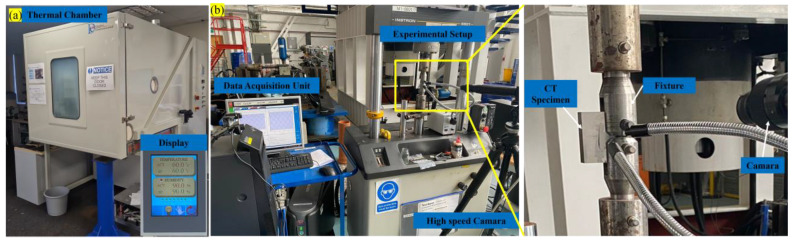
(**a**) Thermal chamber and (**b**) experimental setup.

The fatigue pre-cracked CT specimens were placed in a 3.5% NaCl solution for 3 days at room temperature in order to conduct localised corrosion testing. Subsequently, the specimens were removed, air-dried, and subjected to harsh coastal environmental conditions using a thermal chamber. This chamber simulated temperatures of 20, 40, 60, and 80 °C along with humidity ranging from 40% to 90%, as shown in Figure 2a.

### 2.4. FCGR Experimentation

FCGR investigation on the Al6082 alloy is performed using a servo-hydraulic testing apparatus on a 7 mm thick specimen with a 3 mm initial fatigue pre-crack size. The servo-hydraulic testing machine can apply controlled cyclic loads to the specimen shown in Figure 2b. In the present work (PW), the load ratio (R ratio) considered is 0.1. During the experiment, the specimen is subjected to cyclic loading at 3 Hz. The crack length is continuously monitored and measured regularly using a high-speed camera.

In this experiment, the fatigue life cycles and related crack lengths of the Al6082 alloy were recorded under cyclic loading conditions. The change in crack size over the number of fatigue cycles provided valuable data by which to analyse the FCG behaviour. The FCGR values were also determined under different temperature and humidity conditions.

I Alqathani et al. [5] has established the empirical model to predict the fracture toughness of the Al6082 alloy under combined temperature and humidity conditions as mentioned below:

(1) $$\begin{array}{c} K_{Ic}=46.22-\left(4.687\times 10^{-5}\cdot H^{3}\right)+\left(3.099\times 10^{-6}\cdot TH^{2}\right)-(1.729\times 10^{-5}\cdot T^{2}H)\\ \;\;\;\;\;\;\;\;\;\;\;\;\;\;\;\;\;\;\;\;\;\;\;\;\;\;\;\;\;\;\;+(7.486\times 10^{-7}\cdot T^{3})+(0.011\cdot H^{2})+(0.0005344\cdot TH)\\ \;\;\;\;\;\;\;\;\;\;\;\;\;\;\;\;\;\;\;\;\;\;\;\;\;\;\;\;\;\;\;+(0.001533\cdot T^{2})-(0.8736\cdot H)-(0.04909\cdot T) \end{array}$$

where H is humidity in % and T is temperature in °C. As per the results, the accuracy of the developed model is 98%.

One of the crack growth functions, the Paris–Erdogan model, is given in Equation (2) [41]. The Paris model is limited to describing linear or stable crack growth rates at a specific stress ratio, and modifications have been made to account for different stress ratios. However, Equation (2) was still used to characterise corrosion fatigue crack growth (FCG) behaviour [14].

(2) $$\frac{da}{dN}=C{\left(∆K\right)}^{m}$$

where, $\frac{da}{dN}$ is the crack growth per stress cycle, a is the crack length or size, ∆K is the stress intensity factor (SIF) range, C is the material-specific Paris constant, and m is the material constant.

## 3. Results and Discussion

### 3.1. Fatigue Crack Growth Rate (FCGR)

**Figure 3:**
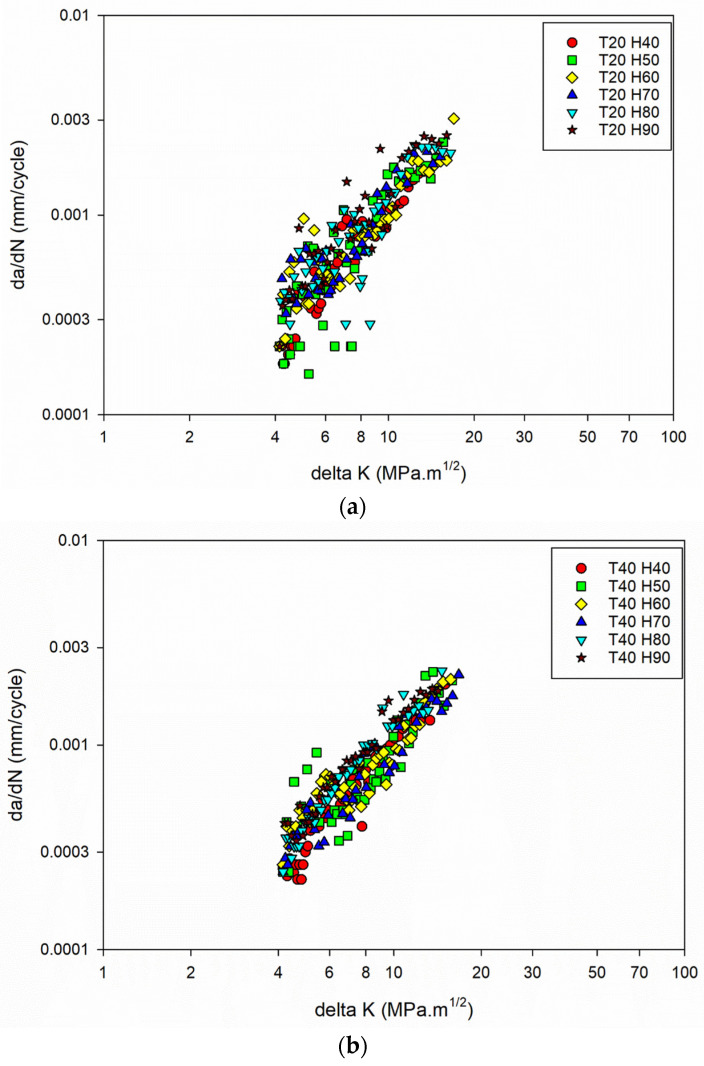

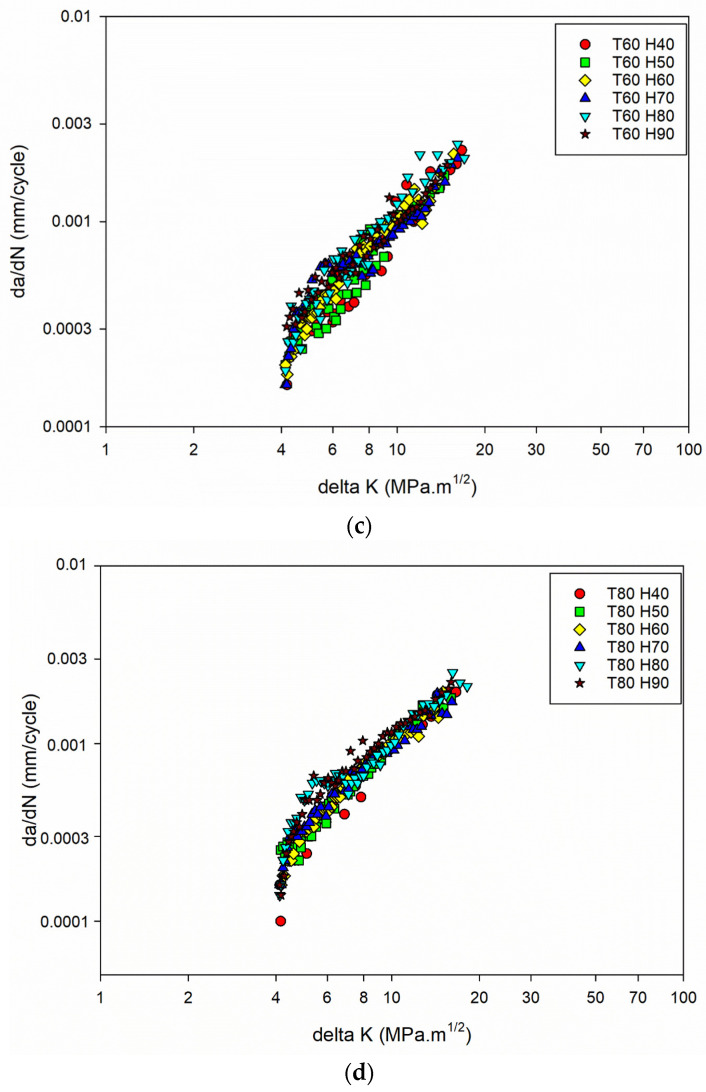
(**a**) FCG results of the Al6082 alloy at a temperature of 20 °C and different humidity levels. (**b**) FCG results of the Al6082 alloy at a temperature of 40 °C and different humidity levels. (**c**) FCG results of the Al6082 alloy at a temperature of 60 °C and different humidity levels. (**d**) FCG results of the Al6082 alloy at a temperature of 80 °C and different humidity levels.

Further understanding of the connection between FCGR (da/dN) and SIF range (ΔK) involved using the obtained data to plot da/dN against ΔK. The plot, often presented on a log–log scale, allows for a linear representation of the FCGR within a specific SIF range. By fitting the experimental data on the da/dN vs. ΔK plot, the FCG coefficients (C and m) can be determined. Figure 3a–d shows the da/dN vs. ΔK plot for different temperature and humidity levels.

**Figure 4:**
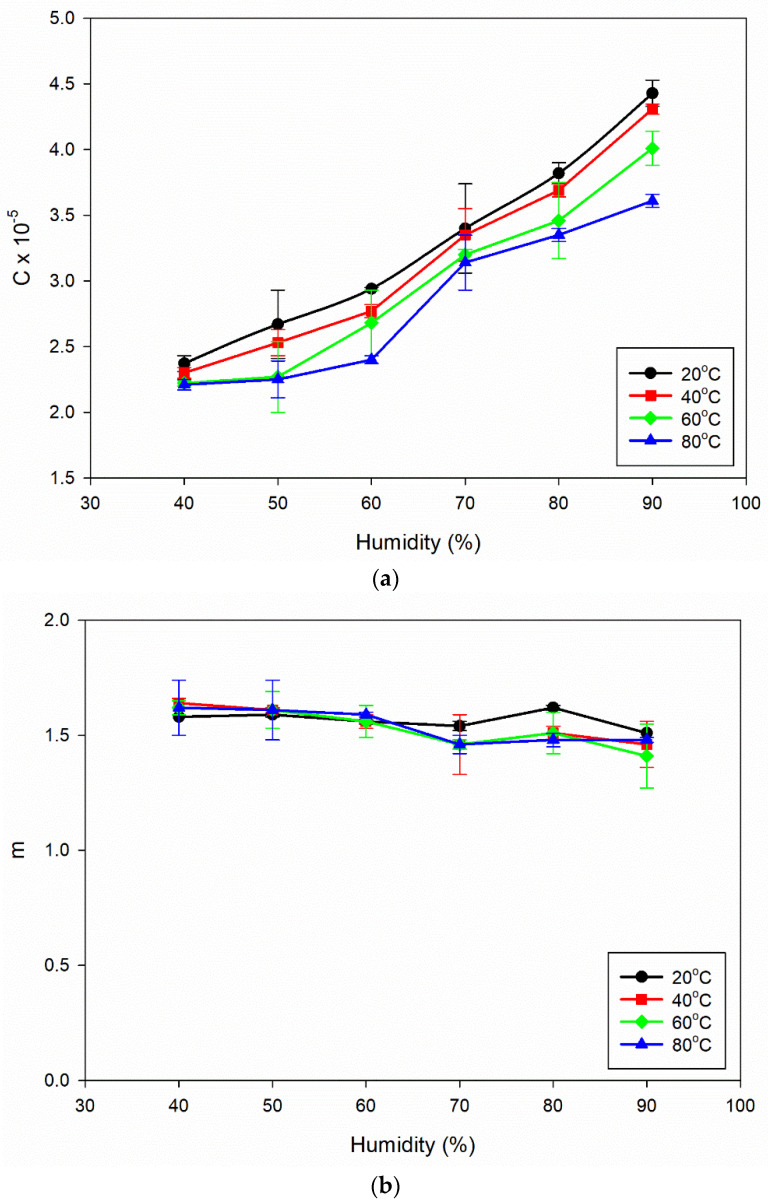

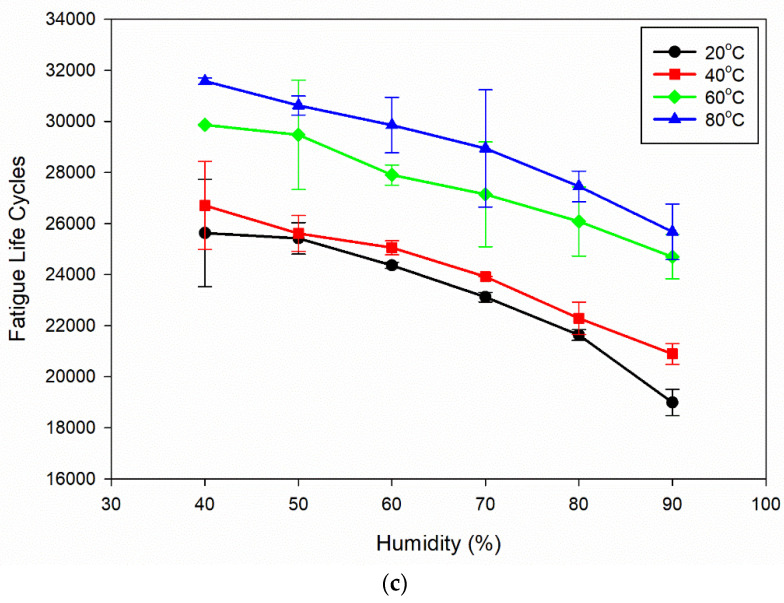
(**a**) Influence of increasing humidity on C values for different temperatures. (**b**) Influence of increasing humidity on m values for different temperatures. (**c**) Influence of increasing humidity on fatigue life cycles for different temperatures.

The da/dN vs. ΔK plot for the power law regime [42] was drawn, with a focus on studying the stable crack growth regime. The exponential growth in the crack size (da/dN), resulting from the power law behaviour, is transformed into a linear connection with the SIF range by plotting the data on a log scale. The values of C and m can be determined using the power law representation and applying regression on the log–log plot of da/dN vs. ΔK. The obtained C and m values are shown in Figure 4a,b.

It is evident from Figure 4a that the value of C increases as the percentage of humidity rises from 40% to 90%. An increase in the C value with rising humidity from 40% to 90% leads to a higher FCGR, indicating faster crack growth rates in the material. This trend suggests that, as the humidity level rises, the resistance to FCG of the Al6082 alloy tends to decrease. In other words, higher humidity conditions can accelerate crack growth rates in the material, indicating a reduced resistance to fatigue crack propagation. From Figure 4a, it can also be observed that an increment in temperature, from 20 °C to 80 °C, leads to a decrement in the values of C. The formation of phase particles at higher temperatures can hinder dislocation movement and slow crack propagation rates. This enhanced resistance to crack growth leads to smaller C values, indicating lower crack growth rates in the material under elevated temperature conditions.

From Figure 4b, a noticeable trend emerges as humidity levels rise, indicating that the m values do not change significantly and remain within the range of 1.41 to 1.64. This indicates that the Al6082 alloy’s crack growth behaviour is relatively consistent under different humidity conditions, and the exponent m in the Paris law equation remains relatively constant within the specified range of humidity values.

The constant m values as humidity increases indicate that the material’s crack growth behaviour remains consistent, and that the da/dN vs. ΔK curves (Figure 3a–d) remain almost the same for different humidity conditions.

The information presented in Figure 4c shows that the fatigue life cycles decrease as the humidity increases from 40% to 90%. As humidity increases from 40% to 90%, the fatigue life cycles decrease, indicating a detrimental effect on the material’s fatigue life. High humidity increases moisture content, accelerating corrosion and promoting crack initiation and propagation. Moisture is corrosive, weakening the material and reducing fatigue life. Additionally, moisture can facilitate hydrogen embrittlement, contributing to crack growth and reduced fatigue life. Increased corrosion and hydrogen embrittlement under higher humidity levels makes the material susceptible to crack propagation and failure under cyclic loading conditions.

### 3.2. Threshold Stress Intensity Factor Range

FCG was investigated by subjecting fatigue pre-cracked Al6082 alloy samples to a 3.5% NaCl solution for 168 h, followed by 72 h of temperature and humidity exposure. The presence of moisture can accelerate corrosion processes, further contributing to crack growth. Thus, the pre-crack fatigue becomes smaller as it gets deeper into the material. Small-crack fatigue thresholds, represented by the threshold SIF range (ΔK_th_), were calculated from the Paris constants C and m, as per the equation mentioned by Robertson and Ritchie [43] and Nestor Perez [44].

(3) ∆Kth=10−6C1m

Using Equation (3), it is possible to predict the threshold SIF range for small cracks by extending the linear stable growth-rate curve downward to a level as low as 10^−6^ mm/cycle. The threshold SIF values have been calculated and are listed in Table 2.

Table 2 presents the threshold SIF values (in units of MPa√m) for the Al6082 alloy under different temperature and humidity levels. The data show that the threshold SIF ranges from 0.37 to 0.61, depending on the specific temperature and humidity combination. These values represent the SIF range below which small cracks do not propagate, signifying the material’s resistance to crack initiation and growth.

From the data, it can be observed that an increment in the temperature leads to an increment in the threshold SIF. This means the material exhibits greater resistance to crack initiation and propagation at higher temperatures. The higher threshold SIF values indicate that the material can withstand higher SIF ranges before cracks propagate. On the other hand, as humidity increases, the threshold SIF decreases. Higher humidity levels make the material more susceptible to crack initiation and growth at lower SIF ranges. This suggests that moisture and increased corrosion potential under humid conditions can reduce the Al6082 alloy’s resistance to crack growth.

The observed range of threshold SIF values, with a low ΔK_th_ value of 0.61 MPa√m, indicates that the Al6082 alloy is susceptible to crack initiation under varying temperature and humidity conditions. The combination of fatigue, corrosion, moisture-assisted crack propagation, and hydrogen embrittlement due to high humidity contributes to the reduced threshold SIF. These factors act together, promoting crack initiation and propagation at relatively low stress levels.

### 3.3. Effect of Temperature and Humidity

The effect of temperature and humidity is discussed in two sections, focusing on the effects of temperature and humidity. This is considered an effective approach to present the findings clearly and in an organised fashion.

#### 3.3.1. Effect of Temperature

In Figure 4a,c, it can be observed that, with an increase in temperature, the value of C decreases, and, at the same time, the fatigue life of the Al6082 alloy increases. This trend indicates that the alloy’s resistance to FCGR improves at higher temperatures. This improved resistance to FCGR at higher temperatures can be attributed to various factors, including reduced corrosion [45], crack closure [46], and the presence of phase particles [47].

*(a)*  *Reduced Corrosion:*

**Figure 5:**
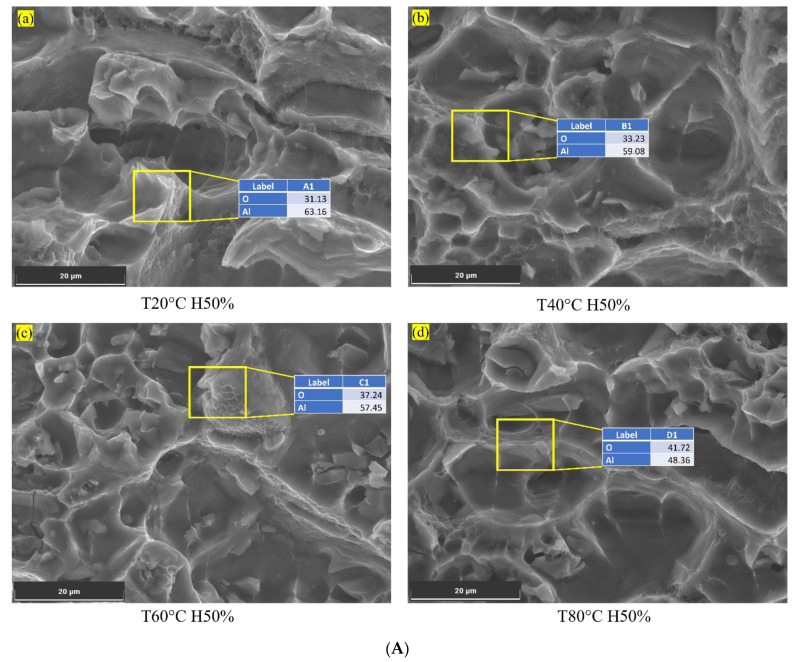

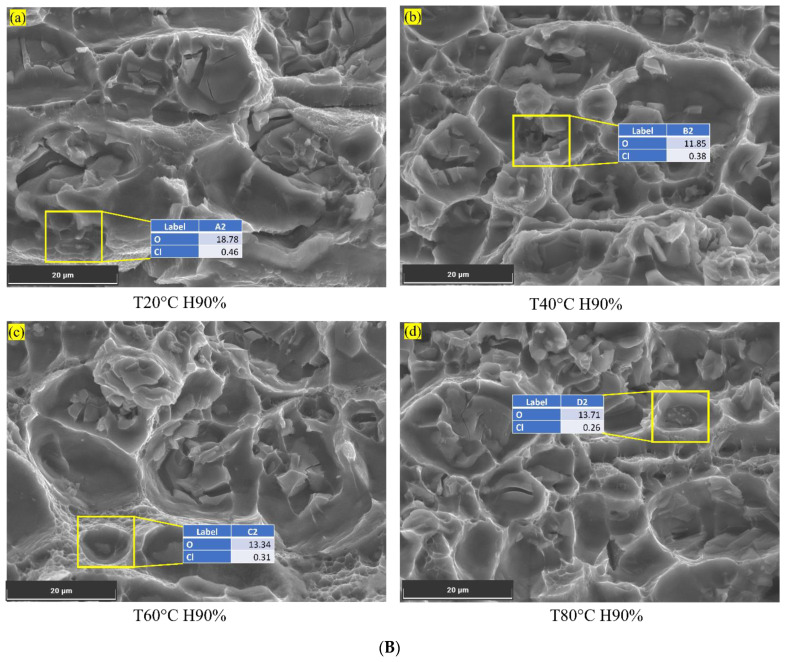
(**A**) Oxide layer on surface of the Al6082 alloy at low humidity and different temperatures. (**B**) Chlorine on the corroded surface of the Al6082 alloy at high humidity and different temperatures.

As temperatures increase, the Al6082 alloy experiences a reduction in the corrosion rate, which can impact crack initiation and growth behaviour. As the temperature increases, the rate of oxide formation accelerates, resulting in a thicker and more protective oxide layer on the surface of the Al6082 alloy, as shown in Figure 5A.

This enhanced oxide layer acts as a barrier [48], effectively slowing down the reaction of the metal with aggressive agents present in the environment. The oxide formation process involves the reaction between aluminium and oxygen in the air, creating a thin layer of aluminium oxide (Al_2_O_3_) on the surface [5]. This oxide layer acts as a physical and chemical barrier, impeding the penetration of corrosive agents in the environment, such as moisture, chlorides, or other chemicals. It hinders their access to the underlying aluminium metal, reducing corrosion initiation and propagation chances. The oxide layer effectively shields the metal from direct contact with the aggressive agents, minimizing the electrochemical reactions that drive the corrosion process.

Moreover, the more stable oxide layer formed at higher temperatures adheres more firmly to the metal surface, enhancing its resistance to degradation. This increased stability and adhesion make it more difficult for aggressive agents to dislodge or degrade the oxide layer, further reducing the overall corrosion rate.

The data also reveal that the activity of aggressive agents, represented by their respective values (0.46 to 0.26), decreases at higher temperatures, as shown in Figure 5. The reduced activity of aggressive agents, such as chlorine, at increased temperatures contributes to the overall decrease in corrosion rate. This increases the corrosion resistance and helps the Al6082 alloy minimise crack initiation and growth.

*(b)*  *Crack Closure:*

**Figure 6:**
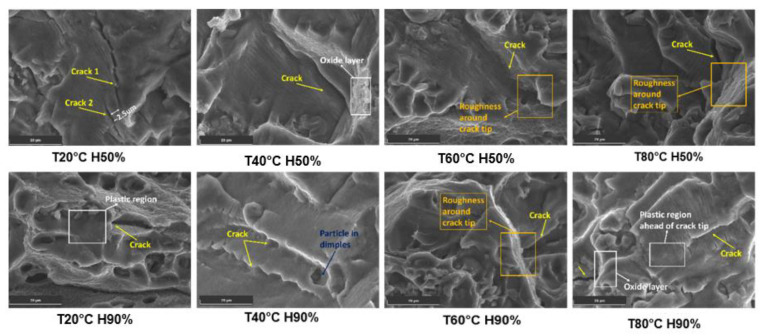
Crack closure mechanisms observed in different conditions. Scale bar 20 μm.

As temperatures increase, the Al6082 alloy undergoes the formation of an oxide layer, develops the presence of phase particles in dimples, and plastic deformation ahead of the crack tip, among other mechanisms [49]. These factors contribute to the phenomenon of crack closure. In Figure 6, SEM images reveal many similar observations which collectively suggest the occurrence of crack closure during certain stages of the loading cycle.

The roughness around the crack tip leads to interlocking and meshing of the crack surfaces, causing crack closure and slowing down FCGR [50]. Additionally, particles in dimples near the crack obstruct the crack path and contribute to crack closure effects. The plastic deformation ahead of the crack tip creates a resistant zone, leading to crack closure and influencing FCGR. Moreover, the oxide layer acts as a protective barrier, hindering aggressive agents’ penetration and further promoting crack closure effects, thus enhancing resistance to FCGR. Additionally, the proximity of two nearby cracks can lead to their interaction and change the direction of the crack, resulting in crack closure behaviour and reducing FCGR.

*(c)*  *Presence of Phase Particles:*

**Figure 7:**
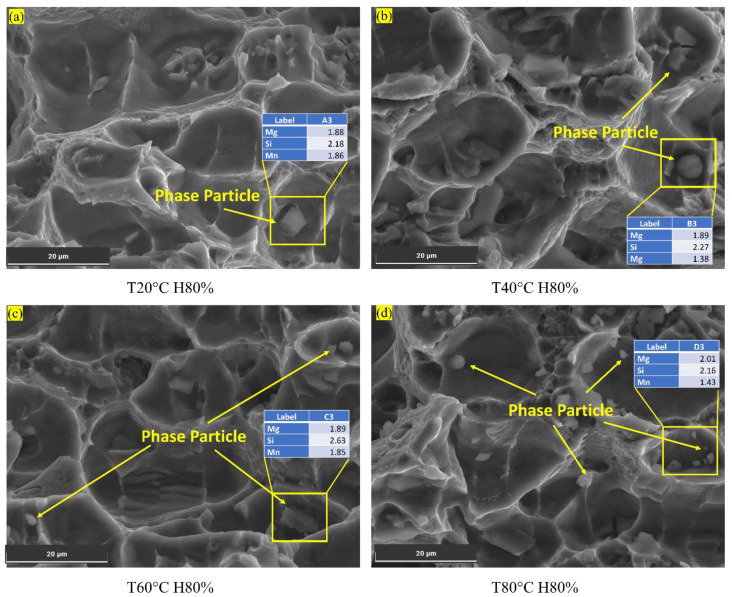
Phase particles observed in different temperature conditions.

As the temperature rises from 20 °C to 80 °C, the Al6082 alloy’s percentage elongation also increases, resulting in improved ductility and enhanced resistance to FCGR. The samples subjected to testing at 60 °C and 80 °C exhibited noticeable phase particles, whereas their presence was considerably diminished at 20 °C and 40 °C (Figure 7a,b). This suggests that phase particle formation primarily occurs at temperatures exceeding 60 °C. Thus, forming phase particles in the Al6082 alloy’s cracked surface decreases crack growth, reducing FCGR [51].

Upon reaching a temperature of 70 °C and above and then cooling, elements such as Mg within the Al6082 alloy tend to aggregate, creating stable particles referred to as precipitates, specifically the Mg_2_Si intermetallic compound [5]. These formed precipitates effectively impede the motion of dislocations, thereby augmenting the material’s mechanical strength. Furthermore, they function as barriers, impeding the ingress of chlorides into the Al6082 alloy.

At room temperature, dislocation motion is low. It increases rapidly from 30 °C to 60 °C but slows down after 60 °C. An increment in temperature from 60 °C to 80 °C minimally impacts resistance to FCG in the Al6082 alloy due to reduced dislocation motion. This reduction allows phase particles to act as effective barriers, regulating void formation, reducing crack nucleation at the crack tip, and slowing down the crack propagation, increasing resistance to fatigue crack growth.

#### 3.3.2. Effect of Humidity

In Figure 4a,c, we can see that, as humidity levels rise, the value of C increases, and, at the same time, the fatigue life of the Al6082 alloy decreases. This trend indicates that the resistance to FCGR of the Al6082 alloy decreases at higher humidity conditions. This decrement in resistance to FCGR at higher humidity conditions can be attributed to various factors, including corrosion [48], moisture-assisted crack propagation [51,52], and hydrogen embrittlement [28].

*(a)*  *Corrosion/Corrosion Fatigue:*

Varying humidity levels can significantly influence the corrosion process in the Al6082 alloy and subsequently impact crack initiation and growth. The presence of chloride ions, which increase with higher humidity levels, is critical in initiating aggressive localized corrosion on the metal surface, such as pitting corrosion.

**Figure 8:**
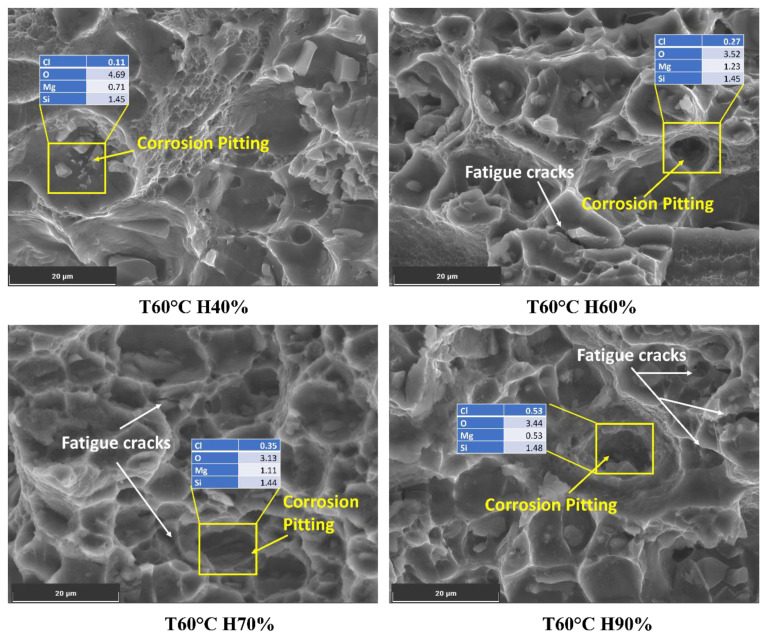
Chloride on the corroded surface of the Al6082 alloy at different humidity conditions.

At 60 °C and 40% humidity, the chloride content is relatively low (0.11%), which may result in minimal localized corrosion. As humidity increases to 60%, 70%, and 90%, the chloride content rises to 0.27%, 0.35%, and 0.53%, respectively, as shown in Figure 8. The presence of chloride ions in these pits facilitates crack initiation and accelerates crack growth rates. As the crack propagates, it encounters varying microstructures, including the phase particles present in the Al6082 alloy. The reduced O value from 4% to 3% with increasing humidity indicates a more reduced oxide layer caused by an increment in the chloride ions. Furthermore, the observed decrease in Mg and Si levels below their actual values suggests that these alloying elements may participate in corrosion processes, leading to material degradation and a reduction in corrosion resistance. This promotes corrosion processes and further leads to accelerated corrosion and crack initiation.

This increase in chloride content significantly enhances the potential for localized corrosion, particularly in pitting [48]. The synergistic effects between humidity and corrosion can lead to corrosion fatigue in the Al6082 alloy. The formation of corrosion pits and cyclic loading during FCGR testing can serve as initiation sites for fatigue cracks. The cyclic stresses applied to the material can open and close the cracks, accelerating the transport of corrosive agents to the crack tip and enhancing crack growth.

*(b)*  *Moisture-Assisted Crack Propagation:*

**Figure 9:**
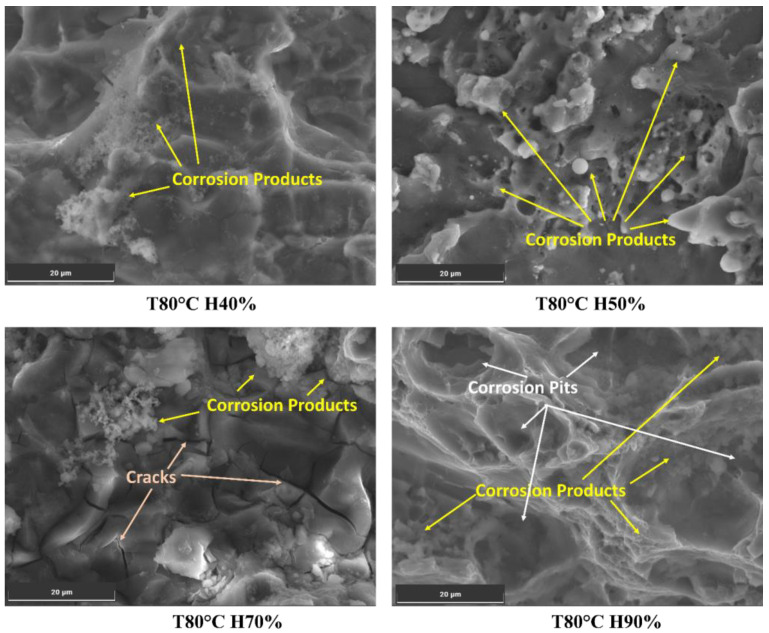
Formation of corrosion products on the surface of the Al6082 alloy at different humidity conditions.

When a material is exposed to higher humidity levels, the absorption and diffusion of moisture into it promote the formation of a thin electrolyte layer (TEL) [53], this can be a thin water layer or a moisture film on its surface. This micro-environment can create localized areas conducive to corrosion, especially in the presence of chloride ions [54]. This enhances the localized corrosion process, leading to the degradation of the material in the vicinity of the crack tip. As a result, the presence of moisture, in combination with corrosion products (oxides, hydroxides, or chloride compounds) [55], further promotes localized corrosion and accelerates crack growth, as shown in Figure 9. The moisture-driven corrosion process weakens the material, making it more susceptible to crack propagation. The synergy between moisture and corrosion products significantly influences crack growth. This might reduce fatigue life and compromise the structural integrity of the Al6082 alloy exposed to such environments.

At low humidity conditions (40%), the reduced moisture on the material’s surface slows down the corrosion process, resulting in a less aggressive corrosion rate than in higher humidity conditions. Typical corrosion products that can form under such conditions include oxides and hydroxides [55]. At high humidity conditions (70% and above), the abundance of moisture on the material’s surface creates a more conducive environment for corrosion, further enhancing forms of localized corrosion, such as pitting corrosion, due to the higher potential for chloride-induced corrosion. Additionally, chloride compounds, such as aluminium chloride, may form in the presence of chloride ions, contributing to the corrosive attack on the aluminium surface. These factors can lead to the formation of corrosion-induced cracks and corrosion fatigue cracks. The combination of high humidity and the presence of chloride ions and corrosive agents may further compromise the material’s structural integrity and decrease the material’s resistance to FCG.

*(c)*  *Hydrogen Embrittlement:*

**Figure 10:**
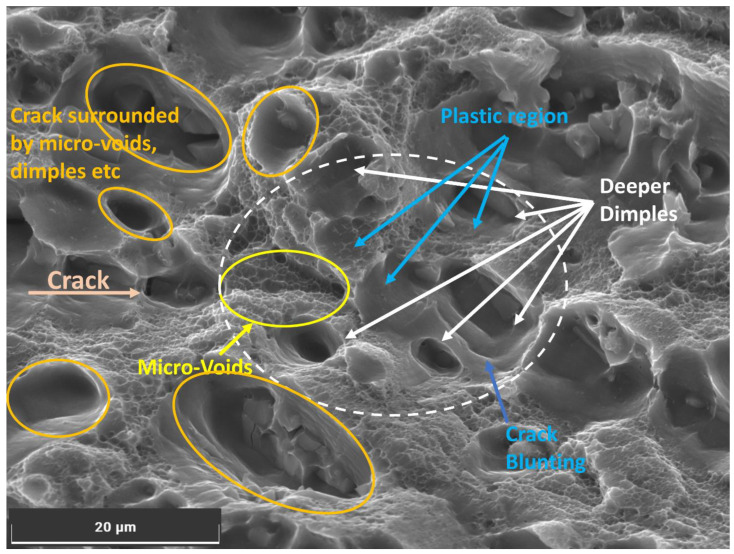
Hydrogen embrittlement mechanism observed at a humidity of 90% and temperature of 20 °C.

As humidity increases, aluminium becomes more susceptible to hydrogen embrittlement, reducing its ductility and toughness and promoting a transition from ductile to brittle fracture behaviour [28]. The observed voids in the material microstructure, shown in Figure 10, indicate hydrogen-induced cracking, where hydrogen accumulates on the surface of the exposed portion of the Al6082 alloy and within the lattice, causing localized weakening and void formation [5]. Void formation indicates hydrogen-induced cracking, while deeper dimples and crack blunting are characteristic of reduced flexibility due to hydrogen diffusion.

These hydrogen embrittlement effects extend beyond the crack tip region, influencing the material’s mechanical behaviour. As humidity increases, the material’s crack growth behaviour becomes more influenced by hydrogen-induced embrittlement, leading to an accelerated crack growth rate. The presence of hydrogen in the material can lower the threshold for crack initiation and promote faster crack propagation, reducing the material’s fatigue life and overall resistance to crack growth. The combined impact of hydrogen embrittlement (HE) contributes to the observed reduction in aluminium’s resistance to fatigue crack growth at higher humidity levels.

**Figure 11:**
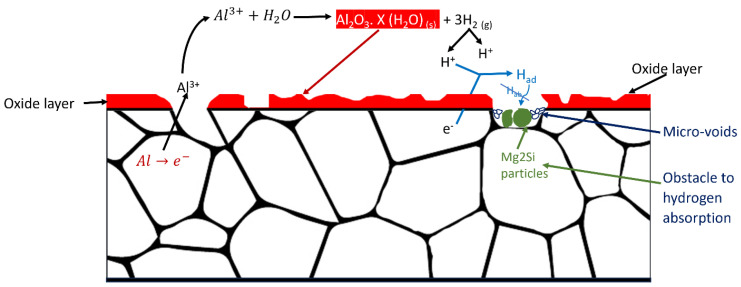
Obstacle hydrogen embrittlement in the Al6082 alloy.

Hydrogen embrittlement (HE) is common in various aluminium alloy series when exposed to humid environments [28]. However, the HE sensitivity is generally low in the Al6xxx series. Despite this, certain Al6xxx series alloys, such as Al6082, remain susceptible to HE even under high humidity conditions [5]. At high temperatures, particularly at 80 °C and high humidity, secondary phase particle formation, such as Mg2Si, is likely in the alloy microstructure. These particles accumulate within the micro-void regions, impeding hydrogen diffusion activity [56]. As a result, hydrogen absorption at the Al6082 alloy’s surface is reduced, as illustrated in Figure 11.

Figure 7d displays the microstructure of the Al6082 alloy at 80 °C and 80% humidity, revealing the presence of phase particles. The energy dispersive X-ray spectroscopy (EDS) analysis of these particles indicates that their composition includes Mg and Si, with values of 2.01 and 2.16, respectively. These EDS results suggest that the detected elements (Mg and Si) are not inherent to the Al6082 alloy itself but rather represent precipitated elements formed under the specific conditions of 80 °C and 80% humidity. The presence of precipitated phase particles in the Al6082 alloy significantly influences its resistance to hydrogen embrittlement, corrosion, and FCGR.

### 3.4. Striation Spaces

During stage II of FCG, the distance separating fatigue striations is equal to the rate at which the fatigue crack expands within a single cycle [57,58,59]. As the crack propagates steadily during each fatigue cycle, it leaves behind a characteristic pattern of striations on the fracture surface. The striation spacing is, therefore, a direct representation of the FCGR under stable crack growth conditions [60].

**Figure 12:**
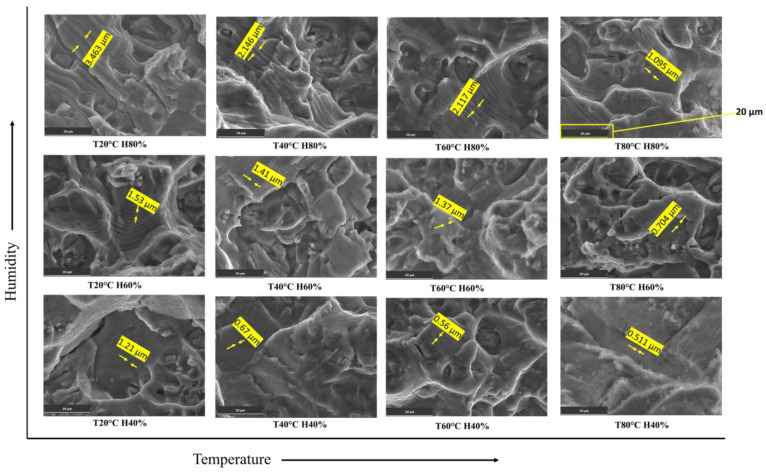
Striations spaces for the Al6082 alloy with varying temperature and humidity.

As humidity increases from 40% to 90%, the environmental conditions become more conducive to corrosion and crack propagation. This can lead to an acceleration in the FCGR of the material. The faster crack growth rate means that the crack propagates over a larger distance during each fatigue cycle, resulting in wider spacing between individual striations on the fractured surface, as shown in Figure 12. The Paris constant (C) is a material-specific parameter in the Paris law equation, which relates the crack growth rate to the SIF range (ΔK). As the FCGR increases with higher humidity, the C also increases to account for the faster crack growth under these environmental conditions. This implies that the Al6082 alloy’s resistance to FCG decreases as humidity increases.

Therefore, the experimental observation of increased striation spacing with increasing humidity is consistent with the higher FCGR and larger C values. The combination of these factors indicates that, as humidity increases, the material’s FCGR becomes more rapid, leading to wider spacing between the striations on the fractured surface.

### 3.5. Crack Propagation Path

**Figure 13:**
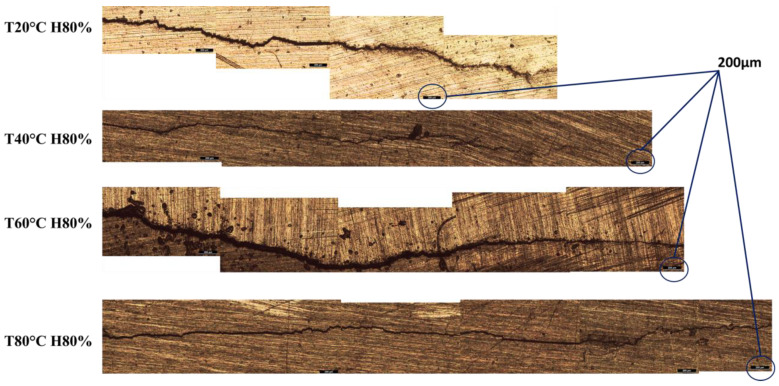
Crack propagation path of the Al6082 alloy at different temperatures (Magnification: 200 μm).

The study of crack propagation paths at different temperatures (ranging from 20 to 80 °C) under 80% humidity has revealed a consistent trend, i.e., that the fatigue cracks propagate along relatively straight paths [61], as shown in Figure 13.

This observation indicates that the fatigue cracks growth behaviour in the Al6082 alloy remains relatively stable across the tested temperature range and under specific humidity conditions. The material exhibits a consistent crack propagation pattern, indicating that the environmental conditions, including humidity and temperature, have a relatively uniform influence on the crack propagation mechanism. The consistent and relatively straight crack propagation paths imply that the material’s fatigue behaviour remains predictable and reliable under temperature and humidity conditions.

### 3.6. FCGR Models for Fatigue Life Cycles and C Value

The significance of this study lies in its comprehensive examination of FCGR in an Al6082 alloy under combined temperature and humidity conditions within a coastal environment. By subjecting the material to varying temperature and humidity levels, we have gained valuable insights into the material’s fatigue crack growth behaviour, crack initiation, and propagation mechanisms. This work aims to develop an empirical model to predict fatigue life cycles and the Paris constant C [62]. Because the experimental m value remains nearly constant with an increase in humidity and temperature, the establishment of the empirical model is not within the scope of this paper.

This study employs a novel curve fitting technique to develop an empirical model that predicts fatigue life cycles and the Paris constant C for the Al6082 alloy under various temperature and humidity variations within a coastal environment. The curve fitting process is applied to the experimental data points to identify the mathematical relationship that best represents the correlation between fatigue life cycles and crack growth rates with temperature and humidity.

**Figure 14:**
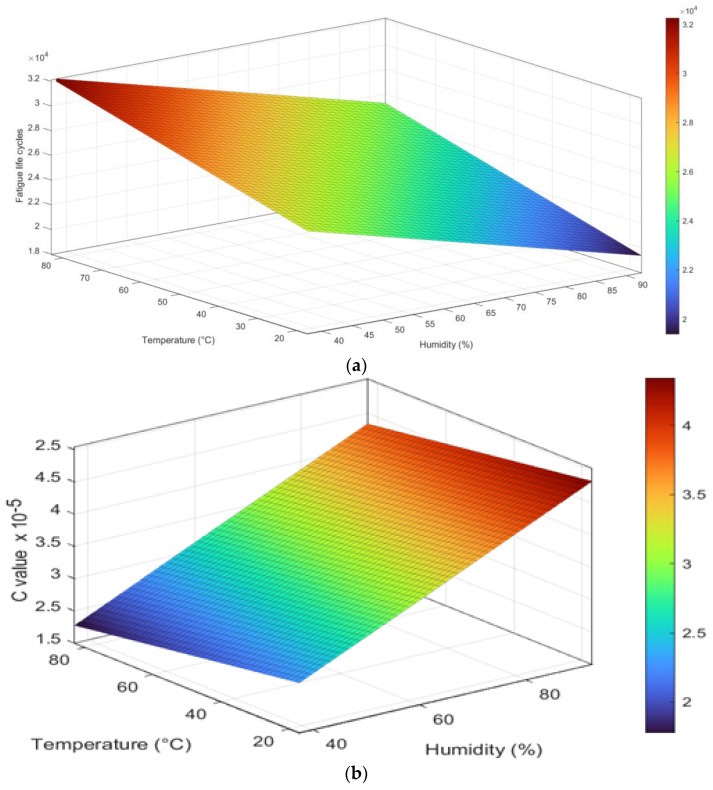
(**a**) Curve fitting shows linear variation for fatigue life cycles with varying temperature and humidity. (**b**) Curve fitting shows linear variation for Paris constant C with varying temperature and humidity.

A 3D scatter plot, as shown in Figure 14a, has been constructed to visualize the connection between temperature, humidity, and fatigue life cycles in the Al6082 alloy. The scatter plot displays data points in a three-dimensional space, where each point represents a specific combination of temperature, humidity, and the corresponding fatigue life cycles obtained from the experimental tests. The empirical equation derived through regression analysis is expressed in Equation (4):

Fatigue life cycle = 29597 + 89.1.T − 126.8.H(4)

The equation indicates that the fatigue life cycles of the material are influenced by both temperature and humidity. A positive coefficient for T suggests that an increase in temperature is associated with an increase in the predicted fatigue life cycles. On the other hand, a negative coefficient for H implies that an increase in humidity leads to a decrease in the predicted fatigue life cycles.

Similarly, a 3D scatter plot, as shown in Figure 14b, has been constructed to visualize the relationship between temperature, humidity, and Paris constant C in the Al6082 alloy. The Paris constant C quantifies how quickly a crack grows under cyclic loading conditions for the Al6082 alloy. The empirical equation derived through regression analysis is expressed in Equation (5):

Paris constant C = 1.0046 − 0.0076·T + 0.0374·H(5)

The equation represents how temperature and humidity levels influence the Paris constant C. A negative coefficient for T suggests that an increment in temperature reduces the predicted Paris constant C. In contrast, a positive coefficient for H signifies that an increase in humidity is associated with an increase in the predicted Paris constant C. Therefore, as humidity increases, the C value increases, and the material’s resistance to FCGR decreases, and as temperature increases, the C value decreases and the resistance to FCGR increases.

## 4. Correlation and Validation

### 4.1. Correlation of Fracture Toughness and Fatigue Life Cycles

The novelty of this work lies in conducting a comprehensive investigation of the FCGR under combined temperature and humidity conditions in a coastal environment, developing an empirical model for accurate fatigue life prediction, and establishing a correlation between FCGR and fracture toughness models.

The established empirical model of fracture toughness [5] is represented by Equation (1), which enables accurate prediction of fracture toughness for different temperature and humidity conditions.

**Figure 15:**
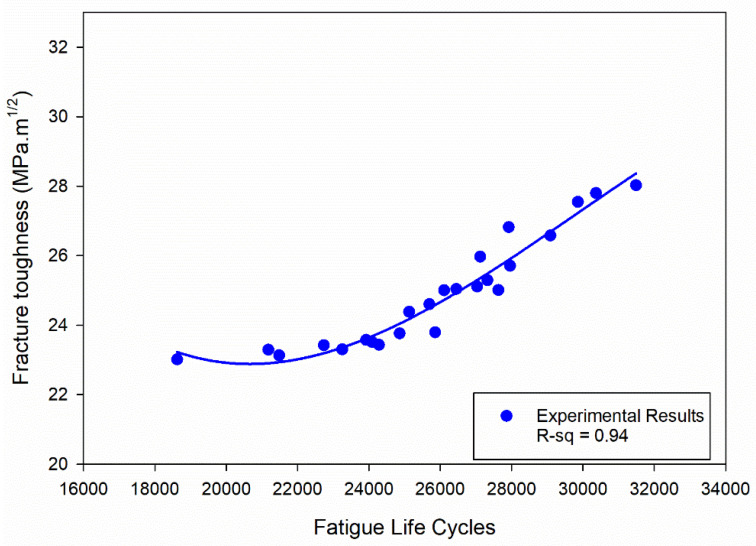
Correlation between fatigue life cycles and fracture toughness.

In Figure 15, the fatigue life cycles found from the FCGR test [PW] and the corresponding fracture toughness values (previous work, [5]) measured for the same material under the same experimental conditions were plotted to examine their correlation using the curve fitting approach. The data points representing the fracture toughness values and the corresponding fatigue life cycles were fitted to a polynomial cubic curve. The selection of a polynomial cubic curve was based on its ability to capture the complexity of the relationship between fracture toughness and fatigue life cycles. An R-squared value of 0.94 indicates that the variation in fatigue life cycles can explain approximately 94% of the variance in fracture toughness.

The empirical model for fracture toughness (K_Ic_) has been developed as Equation (6):

(6) $$K_{Ic}=80.87-0.0068\cdot X+2.5\times 10^{-7}{\cdot X}^{2}-2.83\times 10^{-12}{\cdot X}^{3}$$

where X represents the number of fatigue life cycles. This polynomial cubic curve, with coefficients derived from the curve fitting approach, allows for the accurate prediction of fracture toughness values at varying fatigue life cycles.

Table 3 presents a comparison of fracture toughness values obtained from experimental testing [5] and the values predicted using the regression equation (Equation (6)) developed in this study (PW). It is observed that the maximum error between these two sets of values is within 10%. This error level indicates a relatively good agreement between the empirical model and the experimental data, signifying the effectiveness and accuracy of the developed regression equation in predicting fracture toughness values based on fatigue life cycles.

### 4.2. Validation

The empirical equation for determining fracture toughness (Equation (1)) was derived through fracture toughness testing [5]. Empirical equations for determining fatigue life cycles, the Paris constant C, and the correlation between fracture toughness and fatigue life cycles were derived through FCGR experimentation (PW). These experiments were conducted within the 20–80 °C temperature range and with humidity conditions of 40–90%. A new series of experiments using arbitrarily selected temperature and humidity conditions, such as 30 °C and 85%, 50 °C and 65%, and 70 °C and 55%, and using the Al6082 alloy material was carried out to validate the established empirical equations. The comprehensive analysis of these experimental outcomes has been thoroughly discussed, enhancing the understanding of the empirical models’ validity and their suitability across a wider spectrum of environmental factors.

**Figure 16:**
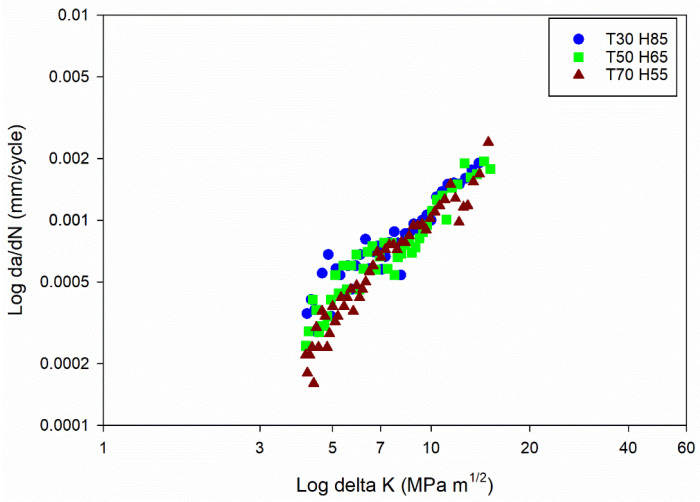
Fatigue crack growth curves for different temperature and humidity conditions.

Figure 16 illustrates the da/dN vs. ΔK plot within the power law regime, considering distinct temperature and humidity conditions as mentioned. The transformation of the exponential growth in crack length (da/dN), arising from power law behaviour, is accomplished by logarithmically scaling the data. This conversion establishes a linear relationship with the SIF range (ΔK). The Paris constant C and exponent m values can be ascertained by employing the power law representation and regression analysis on the logarithmic representation of da/dN vs. ΔK. The resultant C and m values are documented in Table 4 (a). The empirical Equation (5) significantly correlates with the experimental results, demonstrating a maximum error of 8% for the Paris constant C. The m value exhibits minimal variation, remaining within the range of 1.41 to 1.65, as observed in the primary experimental findings.

Likewise, the comparison between experimental and empirical model results of fatigue life cycle values is documented in Table 4 (b). The empirical Equation (4) demonstrates a notable correlation with the experimental results, showcasing a maximum error of 4% for the fatigue life cycle values.

Similarly, the comparison between experimental and empirical model results of fracture toughness values is presented in Table 4 (c). Additionally, empirical Equation (1) displays a substantial correlation with the outcomes derived from empirical Equation (6), indicating a maximum error of 7% for the fracture toughness values.

The strong correlation observed between the experimental and developed models, with a maximum error of 10%, can be attributed to the alignment of the validation conditions (30 °C and 85%, 50 °C and 65%, and 70 °C and 55%) within the broader investigated range of temperature (20–80 °C) and humidity (40–90%). This strategic selection facilitated the verification of the consistency of the equations across diverse environmental scenarios.

## 5. Conclusions

This research investigated the combined influence of temperature and humidity on fatigue crack growth rate in the Al6082 alloy within a coastal environment. The outcomes of this study hold considerable significance for the scientific community, shedding light on the complex relationship between temperature, humidity, and fatigue crack growth rate in the Al6082 alloy within coastal conditions. Through the comprehensive experimental study, the following significant conclusions were drawn:

1. The combined influence of an increase in temperature and humidity levels, in line with coastal environmental conditions, decreases the FCGR resistance of the Al6082 alloy. The corrosion under higher humidity levels reduces the threshold fracture toughness, facilitating crack initiation and propagation at relatively low stress levels. This is evident from the notable decrease in threshold fracture toughness by 27% and the increase in the fatigue crack growth constant C by 34% as temperature and humidity increase.
2. Higher temperature conditions enhance the alloy’s resistance to FCGR by introducing precipitated phase particles, facilitating the formation of an oxide layer, and inducing crack closure. In contrast, heightened humidity conditions diminish the resistance of the Al6082 alloy to FCGR due to escalated corrosion, moisture-assisted crack propagation, and hydrogen embrittlement.
3. The precision of the developed empirical models is remarkable, showcasing an error of less than 10% in predicting Paris constant C, fatigue life cycles, and the relationship between fracture toughness and FCGR. This robust correspondence underscores the models’ reliability for both researchers and engineers. The alignment between experimental and developed models, confirmed through validation experiments, establishes a solid foundation for predicting FCGR in diverse environmental conditions.

## Figures and Tables

**Table 1:** Main elements present in the Al6082 alloy (wt.%) [5].

Element	Mg	Si	Mn	Fe	Zn	Ti	Cu	Cr	Al
wt.%	1.2	1.1	0.9	0.5	0.2	0.2	0.1	0.08	Balance

**Table 2:** Threshold SIF (MPa√m) values of the Al6082 alloy.

Temperature (°C)/Humidity (%)	20	40	60	80
	Threshold SIF (MPa√m)			
**40**	0.58	0.60	0.61	0.61
**50**	0.54	0.56	0.60	0.60
**60**	0.50	0.52	0.53	0.58
**70**	0.45	0.44	0.45	0.46
**80**	0.44	0.42	0.44	0.44
**90**	0.37	0.37	0.37	0.42

**Table 3:** Comparison of fracture toughness values.

Temperature (°C)	Humidity (%)	Fatigue Life Cycles	Fracture Toughness (MPa√m)		Percentage Error
			Experimental [5]	Regression Equation (6)	
20	40	27,122	25.97	23.88	8.0
20	50	25,857	23.79	23.26	2.2
20	60	24,283	23.43	22.64	3.4
20	70	23,251	23.30	22.34	4.1
20	80	21,483	23.13	22.11	4.4
20	90	18,628	23.01	22.66	1.5
40	40	27,923	26.82	24.30	9.4
40	50	26,111	25.00	23.38	6.5
40	60	24,863	23.76	22.85	3.8
40	70	23,921	23.57	22.52	4.4
40	80	22,737	23.42	22.24	5.1
40	90	21,182	23.40	22.11	5.5
60	40	29,857	27.55	25.38	7.9
60	50	27,958	25.71	24.32	5.4
60	60	27,628	25.00	24.15	3.4
60	70	25,698	24.60	23.19	5.7
60	80	25,133	24.38	22.95	5.8
60	90	24,088	24.01	22.58	6.0
80	40	31,488	28.03	26.27	6.3
80	50	30,364	27.80	25.66	7.7
80	60	29,089	26.58	24.95	6.1
80	70	27,325	25.29	23.99	5.2
80	80	27,037	25.11	23.84	5.1
80	90	26,451	25.04	23.54	6.0

**Table 4:** (**a**) Comparison of Paris constant C and m values. (**b**) Comparison of fatigue life cycle values. (**c**) Comparison of fracture toughness values.

**(a)**							
**Sl. No.**	**Temperature** **(°C)**	**Humidity** **(%)**	**Paris Constant C × 10^−5^**				**Paris Constant** **m**
			**Experimental**		**Empirical Equation (5)**	**% Error**	
1	30	85	4.07		3.95	2.95	1.45
2	50	65	3.23		3.05	5.57	1.51
3	70	55	2.33		2.52	7.54	1.63
**(b)**							
**Sl. No.**	**Temperature** **(°C)**	**Humidity** **(%)**		**Fatigue Life Cycles**			
				**Experimental**	**Empirical Equation (4)**	**% Error**	
1	30	85		21,122	21,492	1.72	
2	50	65		25,369	25,810	1.71	
3	70	55		27,863	28,860	3.45	
**(c)**							
**Sl. No.**	**Temperature** **(°C)**	**Humidity** **(%)**		**Fracture Toughness (MPa** **√** **m)**			
				**Empirical Equation (1)**	**Empirical Equation (6)**	**% Error**	
1	30	85		23.29	22.11	5.1	
2	50	65		24.09	23.05	4.3	
3	70	55		26.04	24.27	6.8	

## Data Availability

Data sharing does not apply to this article as no datasets were generated or analysed during the current study.
